# Supplementary material for: Pushing detectability and sensitivity for subtle force to new limits with shrinkable nanochannel structured aerogel
Source: Nat Commun. 2022 Mar 2;13:1119. doi: 10.1038/s41467-022-28760-4 (PMC8891261; doi:10.1038/s41467-022-28760-4)
Supplement: Supplementary file 1 — Supplementary Information [file 41467_2022_28760_MOESM1_ESM.docx]

**Supplementary Information**

**Pushing Detectability and Sensitivity for Subtle Force to New Limits with Shrinkable Nanochannel Structured Aerogel**

Xinlei Shi^1^, Xiangqian Fan^1^, Yinbo Zhu^2^, Yang Liu^1^, Peiqi Wu^1^, Renhui Jiang^3^, Bao Wu^2^, Heng-An Wu^2^, He Zheng^3^, Jianbo Wang^3^, Xinyi Ji^1^, Yongsheng Chen*^4^, Jiajie Liang*^1,4,5^

^1^ School of Materials Science and Engineering, National Institute for Advanced Materials, Nankai University, Tianjin 300350, China.

^2^ CAS Key Laboratory of Mechanical Behavior and Design of Materials, Department of Modern Mechanics, CAS Center for Excellence in Complex System Mechanics, University of Science and Technology of China, Hefei 230027, China

^3^ School of Physics and Technology, Center for Electron Microscopy, MOE Key Laboratory of Artificial Micro- and Nano-structures, and Institute for Advanced Studies, Wuhan University, Wuhan 430072, China

^4^ Key Laboratory of Functional Polymer Materials of Ministry of Education, College of Chemistry, Nankai University, Tianjin 300350, China

^5^ Tianjin Key Laboratory of Metal and Molecule-Based Material Chemistry and Collaborative Innovation Center of Chemical Science and Engineering (Tianjin), Nankai University, Tianjin 300350, China

E-mail: [liang0909@nankai.edu.cn](mailto:liang0909@nankai.edu.cn) (J. Liang), [yschen99@nankai.edu.cn](mailto:yschen99@nankai.edu.cn) (Y. Chen)

**Supplementary Note 1:**

**Calculation of critical stress values.** For MXene aerogels with honeycomb-like cellular structures, the cellular walls can be simplified with two-edge fixed elements in a mechanical model (Supplementary Figure 16). The critical stress (σ_c_) that triggered the bending or buckling of cellular walls was calculated by Equation (1)^1^:

$\sigma_{cr}=\frac{20}{\left( 1+sin\alpha\right)\cos\alpha}\cdot\frac{E}{\left( 1-v^{2} \right)}\cdot\left( \frac{t}{L} \right)^{3}$ (S1)

where α is the structural angle in the microcells (Supplementary Figure 16), ν is the Poisson’s ratio of the multilayer MXene nanosheets (0.23)^2^, *E* represents the Young’s modulus of the MXene (~0.3 TPa)^3^, and *t* and *l* represent the thickness and length of the cellular walls, respectively. As observed from the SEM and HRTEM characterizations, the average values of *l* and *t* were approximately 300 µm and 10 nm, respectively. Accordingly, the critical stress calculated for BBP-MX-AG, ICP-MX-AG, and MX-AG was approximately 0.11 Pa.

**Supplementary Note 2:**

**Molecular Dynamics Simulations.** We performed molecular dynamics (MD) simulations to investigate the deformation and modulus of bottlebrush-like PGPDMS and crosslinked PGPTMS. For PGPDMS, the polymer chains can only crosslink with MXene sheets through chain-end Si-OH groups, whereas PGPTMS chains can also crosslink with each other. Thus, we considered two initial models for these two scenarios, respectively. In the first model, the PGPDMS polymer chains were aligned parallel, and the two ends of each chain were fixed (Supplementary Figure 7a). In the second model, PGPTMS polymer chains are built as a network due to crosslinking, and the two ends of each long chain were fixed (Supplementary Figure 7b). Here, fixing the chain ends represents the crosslinking between the polymer and MXene. After energy minimization of the initial models, both systems were relaxed in the canonical (NVT) ensemble with free boundary conditions. During relaxation, all silicon atoms at the end of the chain were fixed to avoid polymer agglomeration. After relaxation, the two models were compressed to obtain the stress-strain curves and deformation modes. All MD simulations were performed using LAMMPS code^4^. ReaxFF was employed due to its acceptable accuracy and low computational expense compared with methods based on quantum mechanics^5^. More details about the force field used in this work are available in Ref^6^. The relaxed models were established with free boundaries along the *x*-direction and fixed boundaries along the *y*-direction. Reflecting walls were set at the *y* and *z* boundaries to confine the entire system. Compression tests were conducted along the *z*-direction in the NVT ensemble below 300 K. During compression, the displacement along the *x*-direction of all silicon atoms at the end of the chain was limited to zero. The engineering strain rate was 0.001 ps^-1^ in both simulations.

The molecular chains in PGPDMS can deform freely compared with those in the PGPTMS polymer model because the crosslinking points between polymer chains limit their deformation (Supplementary Figure 8); therefore, the modulus of PGPDMS under compression is much smaller than that of PGPTMS. In the snapshots, PGPDMS can be compressed easily because its branched chains and can be curved freely due to a lack of crosslinking points between adjacent chains, whereas the deformation of trimethyl-type chains was severely restricted due to crosslinks between adjacent long chains; therefore, the ultralow modulus and ultrasoft nature of BBP-MX-AG were attributed to the excellent compressibility of bottlebrush-like the polymer.

**Supplementary Note 3:**

**FTIR and XPS characterization.** The covalent crosslinking between MXene and PGPDMS (or PGPTMS) was confirmed by Fourier-transform infrared (FTIR) spectra (Supplementary Figure 12 and 13) and X-ray photoelectron spectroscopy (XPS) (Supplementary Figure 14). Compared with pure MX-AG, three new peaks at 1245, 1197, and 465 cm^-1^ ascribed to Si-CH_2_ and Si-O-Si stretching, and O-Si-O deformation, respectively^7^, appeared in the spectra of BBP-MX-AG (before annealing) and BBP-MX-AG. Compared with the spectrum of BBP-MX-AG (before annealing), a new characteristic Ti-O-Si peak at 942 cm^-1^ appeared in the spectra of BBP-MX-AG and ICP-MX-AG, indicating that the MXene nanosheets were covalently linked with PGPDMS and PGPTMS through Ti-O-Si hetero-linkages in BBP-MX-AG and ICP-MX-AG^7, 8, 9, 10^.

The O 1s spectrum of BBP-MX-AG displays a new peak at a binding energy of 532.3 eV due to Si-O-Si bonds (Supplementary Figure 14). Moreover, compared with BBP-MX-AG (before annealing), a new peak was observed for BBP-MX-AG at a binding energy of 531.7 eV, which was assigned to Ti-O-Si according to previous reports^7, 11^.

**Supplementary Note 4:**

**GPC measurements.** Gel permeation chromatography (GPC) was carried out using an LC20 high-performance liquid chromatography pump (Shimadzu Corporation, Japan), RID-20 differential refraction detector (Shimadzu Corporation, Japan), TSKgel GMPWXL aqueous-phase gel column (TOSOH Corporation, Japan), and HW-2000 GPC chromatographic working station. The mobile phase was 0.1 M NaNO_3_ and 0.06% NaN_3_ aqueous solution. The standard sample was low-PDI polyethylene glycol (PEO). The mobile phase flow rate was 0.6 mL/min, and the column temperature was 35 ℃.

**Supplementary Figures:**


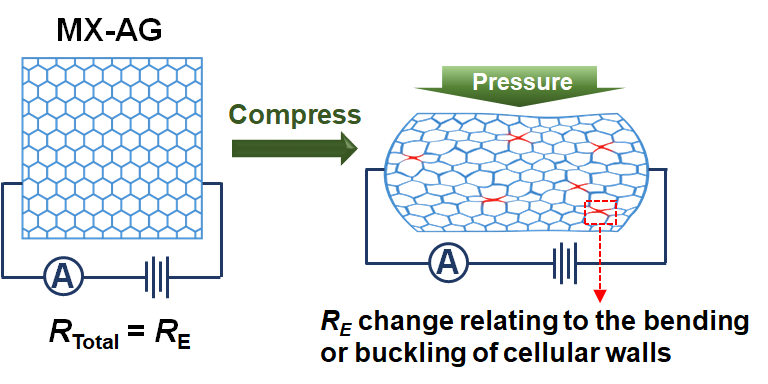


**Supplementary Figure 1.** Piezoresistive sensing mechanism of MX-AG under pressure. The resistance change was mainly induced by the bending or bucking of cellular walls in the aerogel.


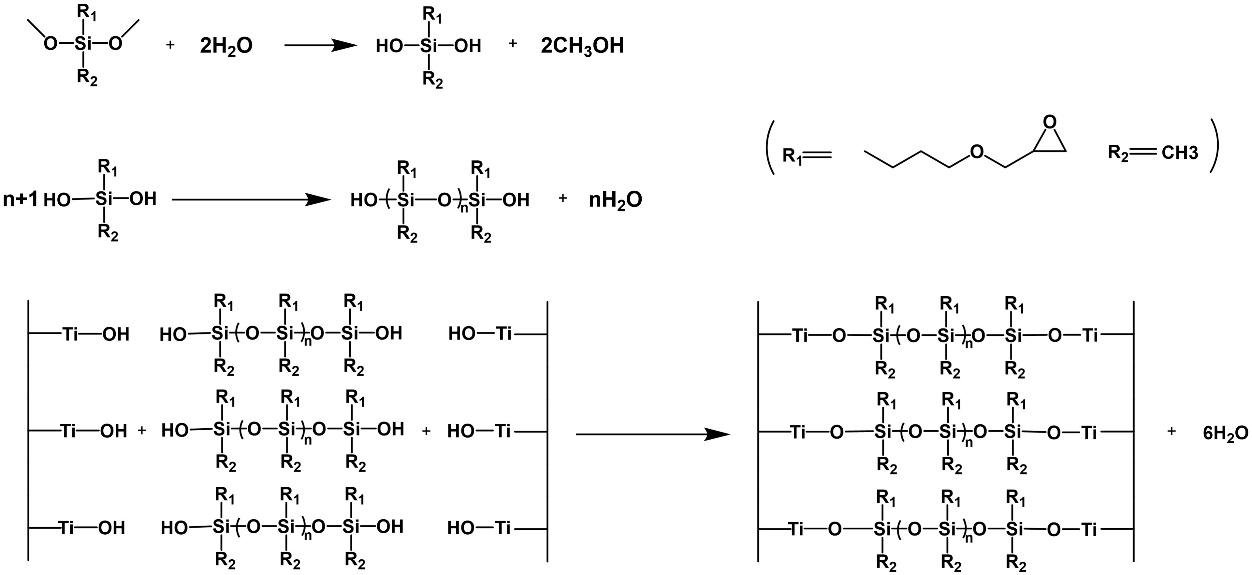


**Supplementary Figure 2**. Synthesis routine of bottlebrush-like PGPDMS intercalated MXene nanochannel structures in BBP-MX-AG.


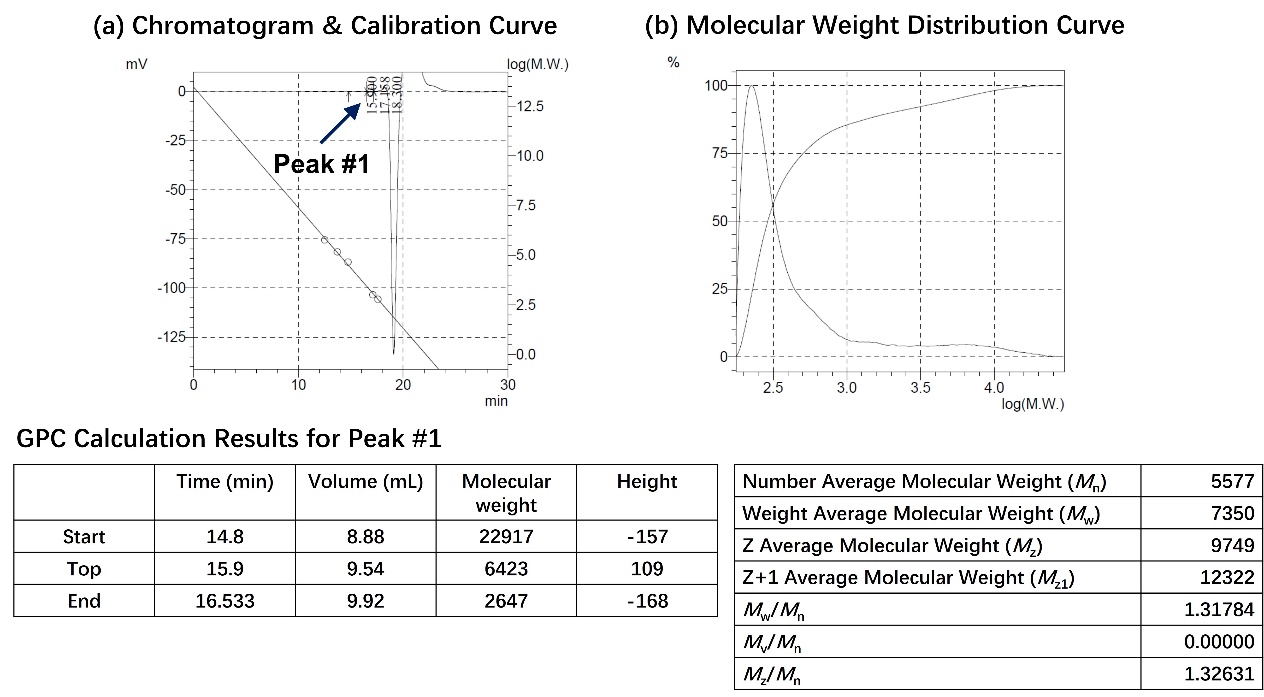


**Supplementary Figure 3**. GPC results for the synthesis of bottlebrush-like PGPDMS.


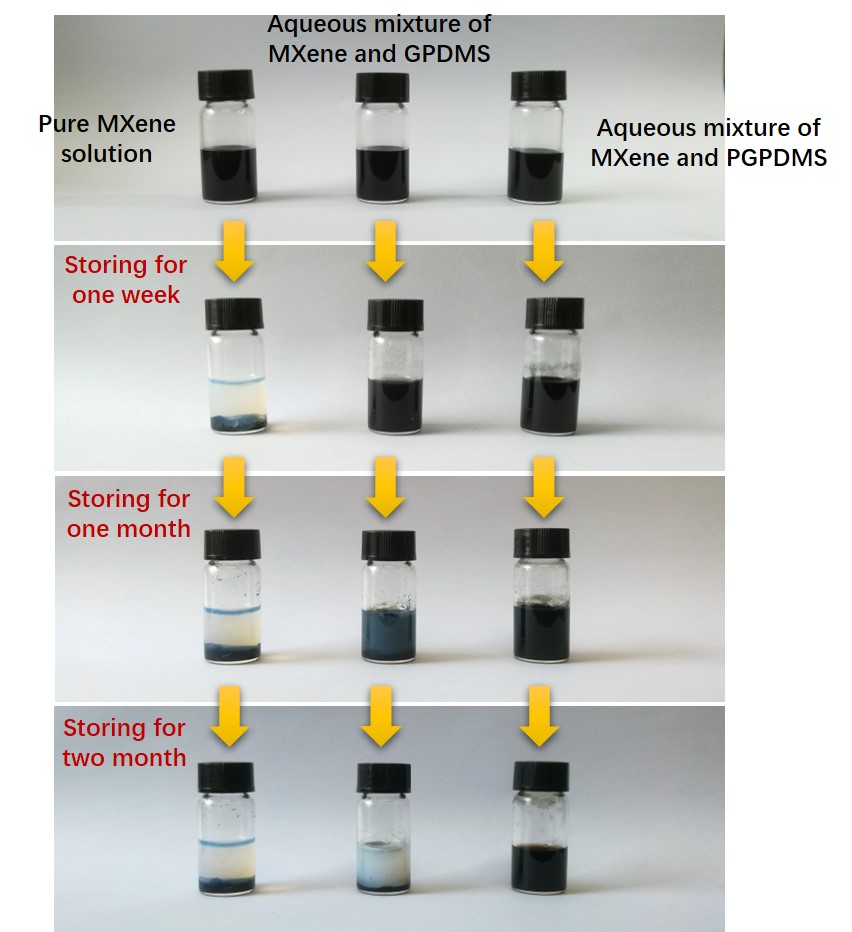


**Supplementary Figure 4.** Comparison of the storage stability of the MXene-based aqueous solution in ambient conditions. Solution samples included a pure MXene solution (20 mg/ml), an aqueous mixture of MXene and GPDMS (MXene: GPDMS = 5:1), and an aqueous mixture of MXene and PGPDMS (obtained from the aqueous mixture of MXene and PGPDMS precursors treated by a hydrothermal process). The pure MXene solution turned white, and abundant precipitates appeared after storage under ambient conditions for one week, indicating severe oxidation and degradation of the MXene. In contrast, partial precipitation occurred in the MXene and GPDMS mixture after storage for one month, and almost no precipitation was observed in the MXene and PGPDMS mixture, even after storage for two months. This indicated that PGPDMS was intercalated into the MXene nanosheets and prevented MXene oxidation and degradation.


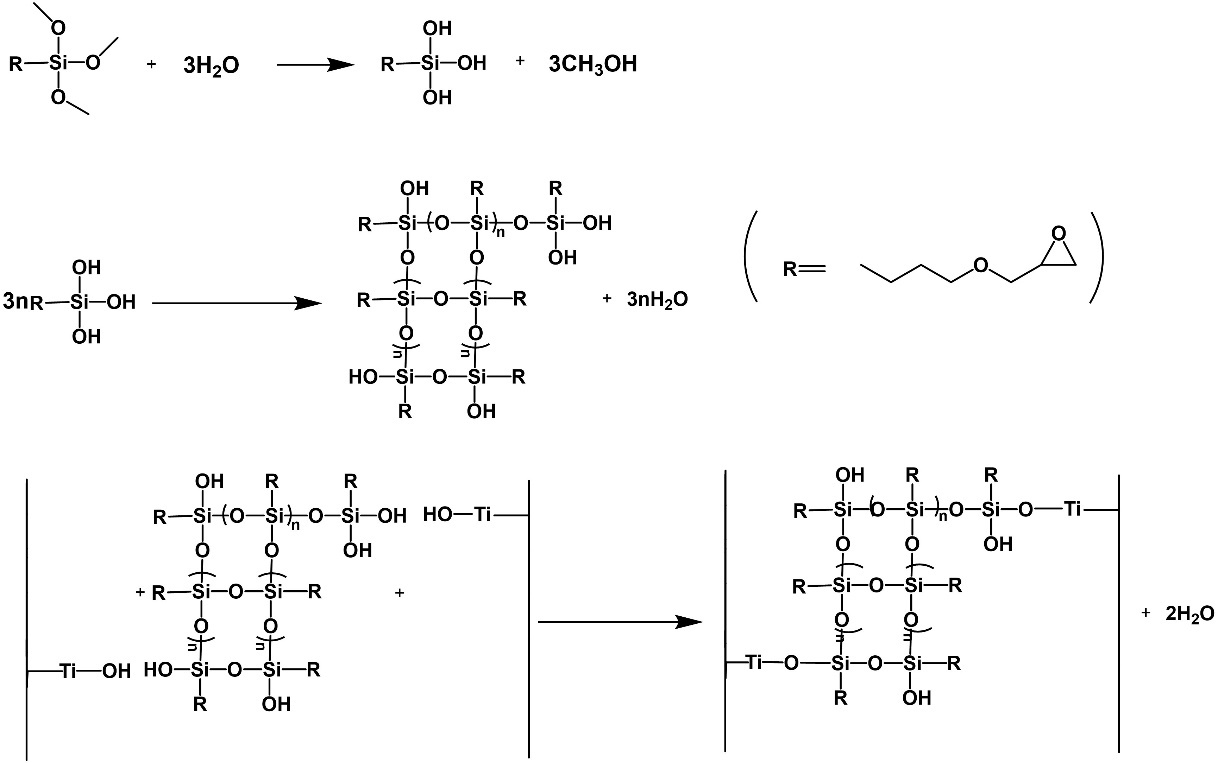


**Supplementary Figure 5.** Synthesis route of interchain-crosslinked PGPTMS intercalated MXene nanochannel structures in ICP-MX-AG.


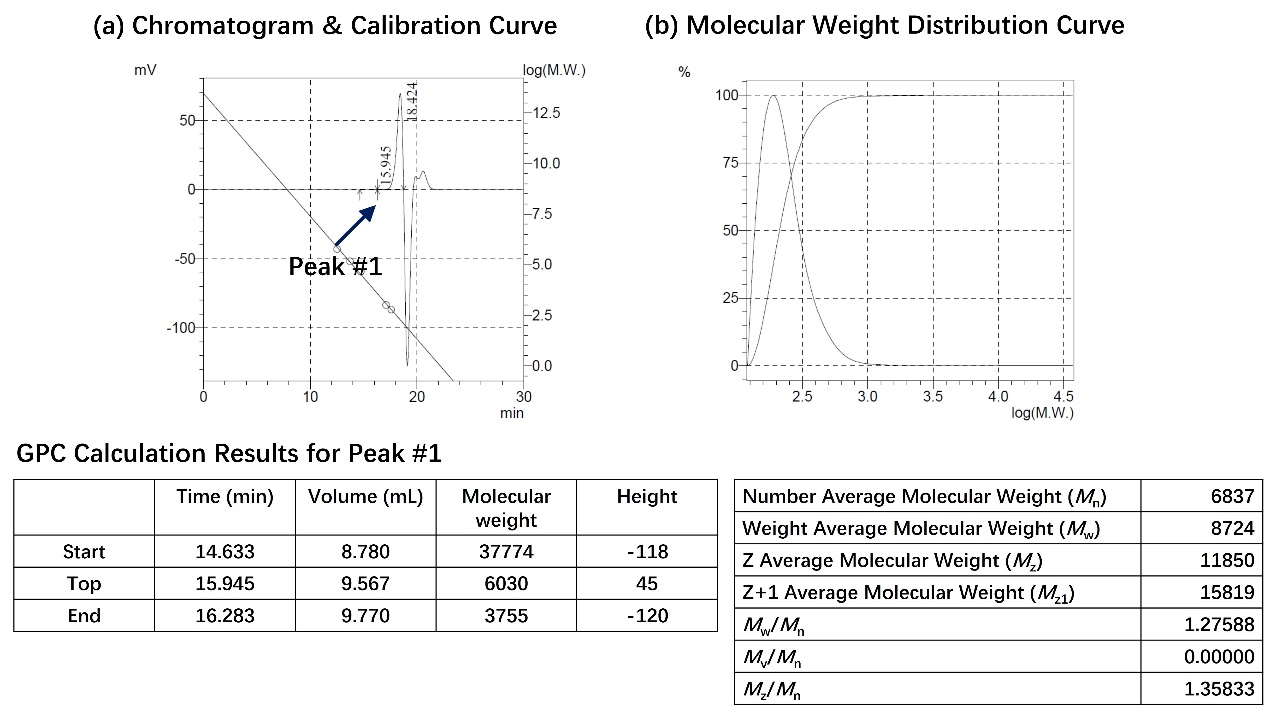


**Supplementary Figure 6**. GPC results for the synthesis of interchain-crosslinked PGPTMS.


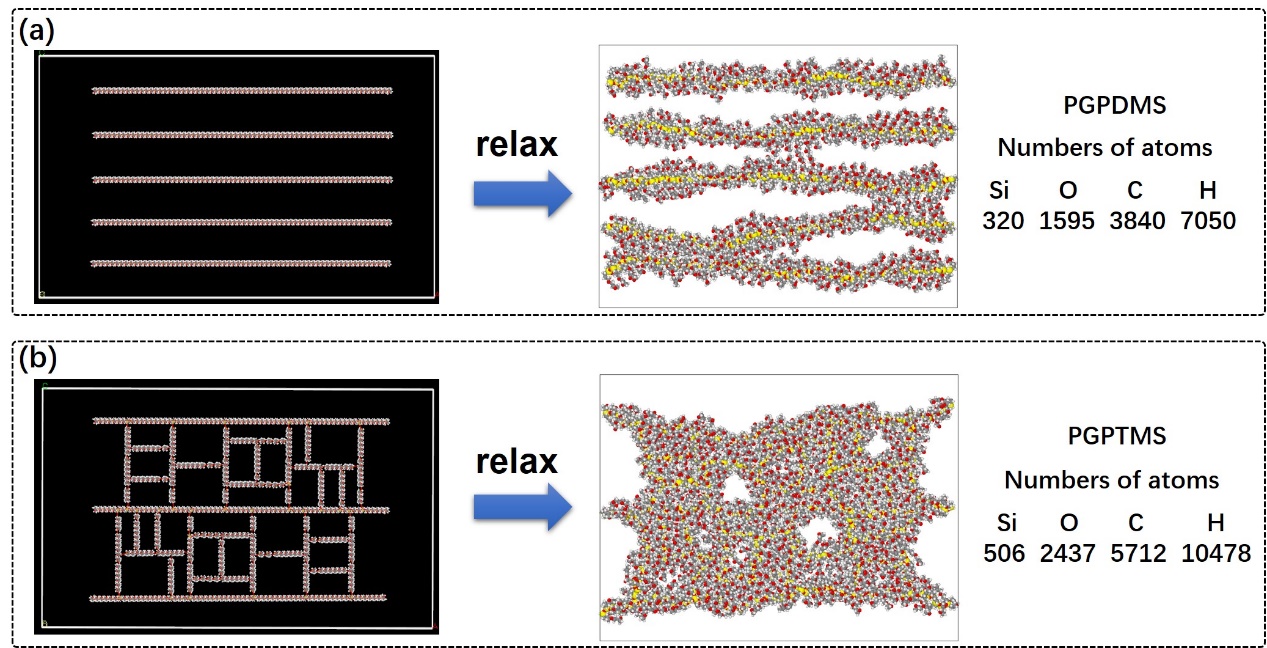


**Supplementary Figure 7.** Models used for molecular dynamics simulations. (a) A single-chain PGPDMS model. (b) A quasi-network model of interchain-crosslinked PGPTMS. The ends of long chains were fixed. The two models were relaxed in the NVT ensemble. The Si, C, O, and H atoms are shown in yellow, grey, red, and white, respectively.


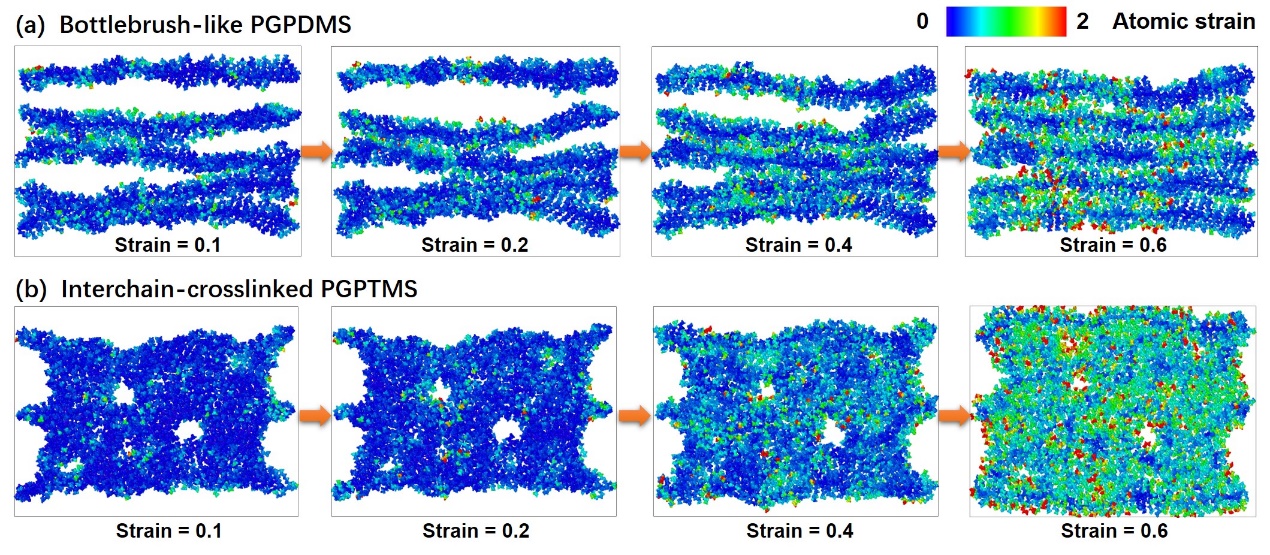


**Supplementary Figure 8.** Molecular dynamic simulations of the atomic configurations under different compressive strains for (a) bottlebrush-like PGPDMS and (b) interchain-crosslinked PGPTMS. The atoms are colored by their atomic strain. During compression, short branched chains in the bottlebrush-like PGPDMS were compressed, while the long main chains were curved, which endowed the bottlebrush-like polymer with excellent compressibility and an ultra-soft nature.


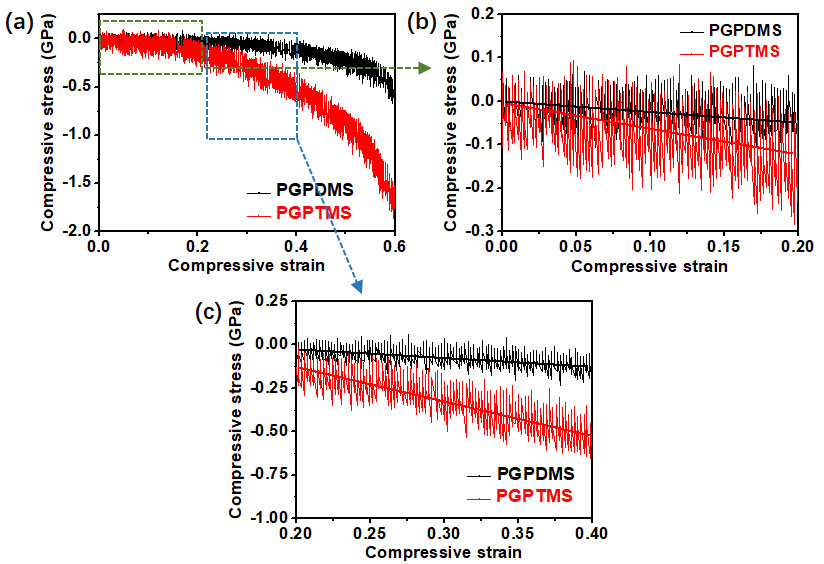


**Supplementary Figure 9.** Simulated compressive stress-strain curves for PGPDMS and PGPTMS in linear fitting strain ranges of (a) 0–0.6, (b) 0–0.2, and (c) 0.2–0.4.


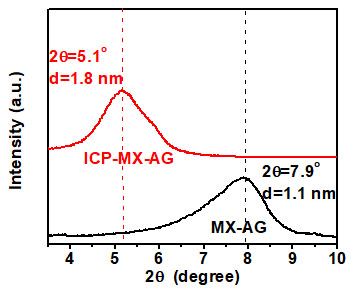


**Supplementary Figure 10**. XRD patterns of ICP-MX-AG and MX-AG. The (0002) peak for ICP-MX-AG displayed a downshift from 7.9^o^ (MX-AG) to 5.1^o^, indicating the interlayer distance of MXene nanosheets increased from about 1.1 nm in MX-AG to 1.8 nm in ICP-MX-AG. This confirms the intercalation of PGPTMS into the MXene interlayer to form parallel nanochannel structures inside the cellular walls of ICP-MX-AG.


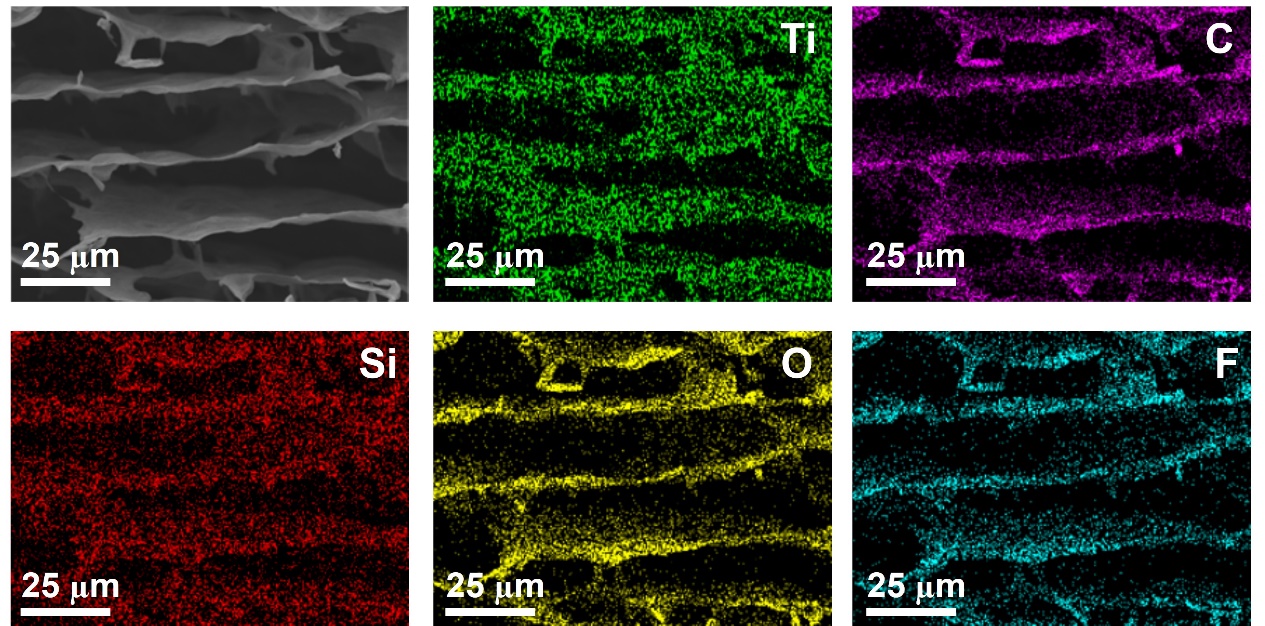


**Supplementary Figure 11.** SEM image and EDS element maps of the scaffold in BBP-MX-AG, indicating the presence of PGPDMS and the spatial distribution of Si atoms (from PGPDMS) over a large area in BBP-MX-AG.


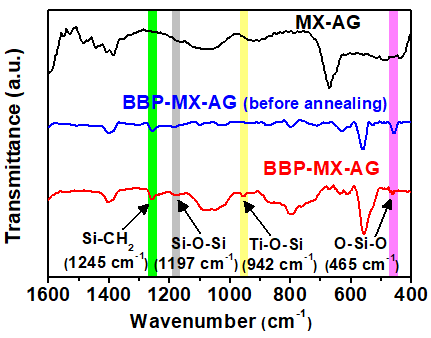


**Supplementary Figure 12.** FT-IR spectra of MX-AG, BBP-MX-AG (before annealing), and BBP-MX-AG.


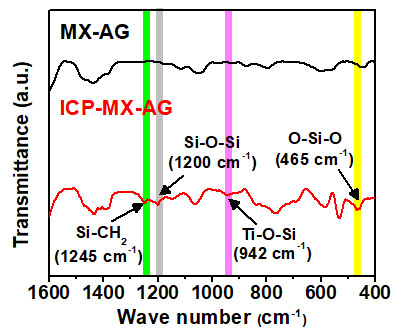


**Supplementary Figure 13.** FT-IR spectra of MX-AG and ICP-MX-AG.


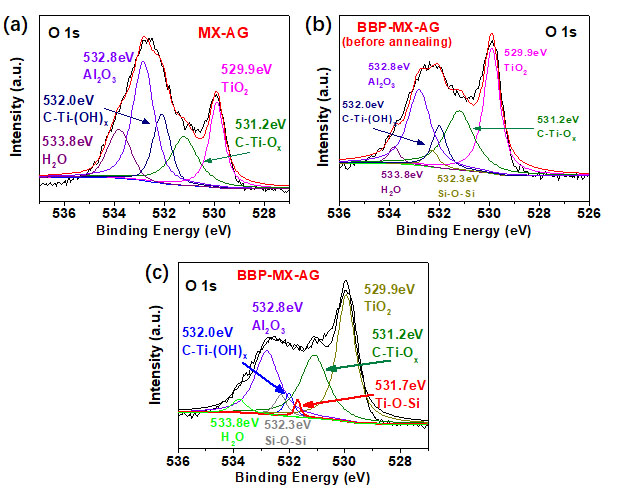


**Supplementary Figure 14.** XPS spectra of (a) MX-AG, (b) BBP-MX-AG (before annealing), and (c) BBP-MX-AG in the O 1s region.


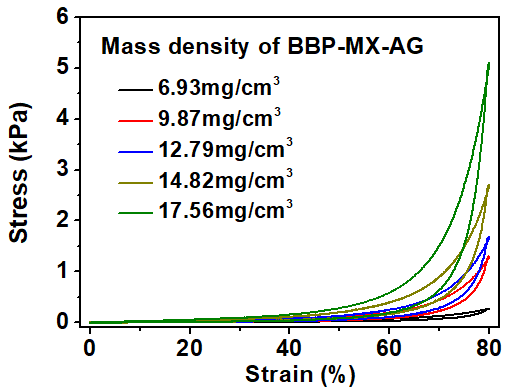


**Supplementary Figure 15.** Compressive stress-strain curves of BBP-MX-AG with various mass densities during compression-release cycles with a maximum strain of 80%.


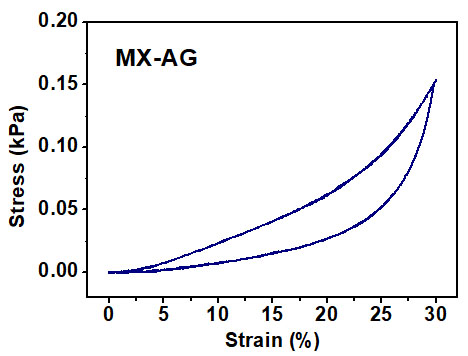


**Supplementary Figure 16.** Compressive stress-strain curve of MX-AG during compression-release cycles with a maximum strain of 30%.


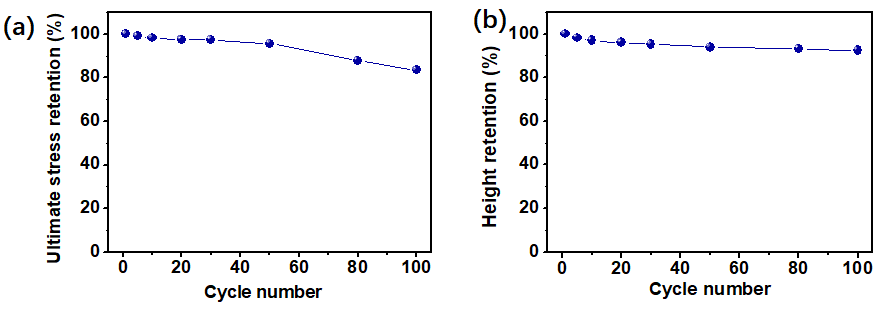


**Supplementary Figure 17.** (a) Ultimate stress retention and (b) height retention of BBP-MX-AG over 100 compression cycles with a compressive strain between 0–80%.


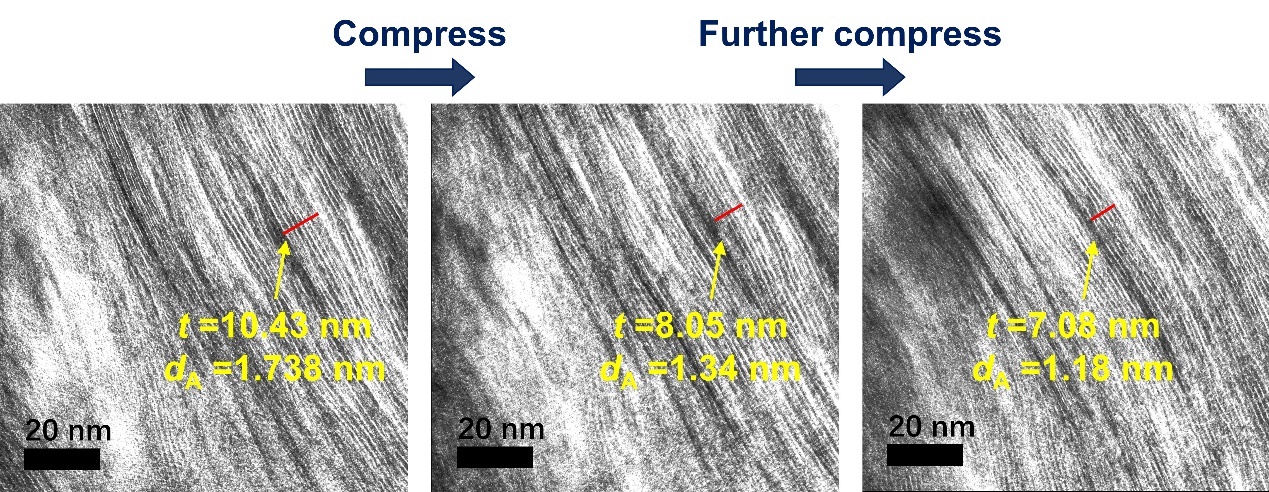


**Supplementary Figure 18.** *In-situ* HRTEM images of the shrinking nanochannels (labeled by red lines) in the cellular walls of BBP-MX-AG. The average distance (*d*_A_) between two MXene layers decreased from an initial value of 1.74 nm to 1.34 nm and 1.18 nm sequentially during the loading process.


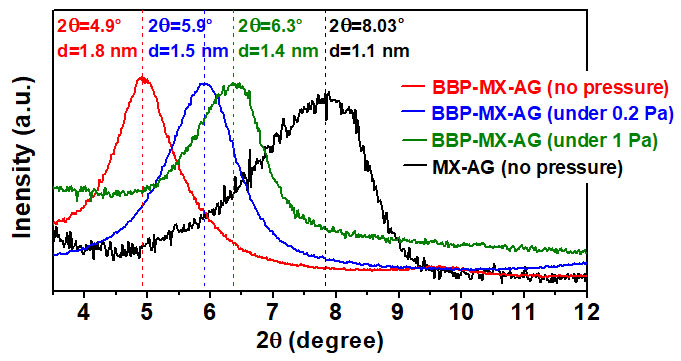


**Supplementary Figure 19.** XRD patterns of MX-AG, BBP-MX-AG without an external force, and BBP-MX-AG under applied pressures of 0.2 Pa and 1 Pa.


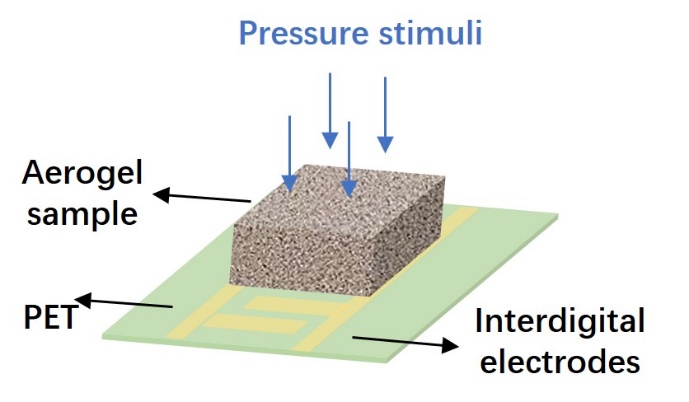


**Supplementary Figure 20.** A monolithic aerogel was assembled into a resistive-type pressure sensor by integrating a monolithic sample on top of an interdigital electrode-coated flexible PET substrate.


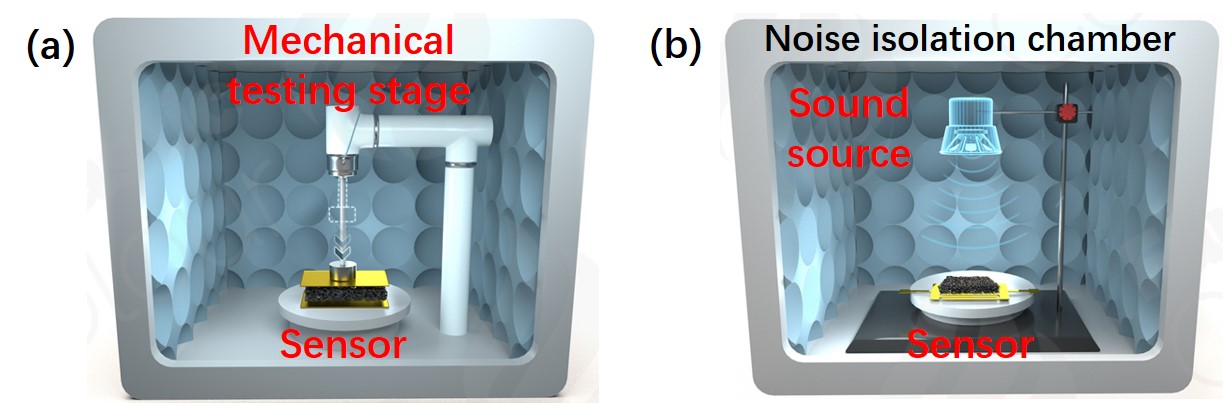


**Supplementary Figure 21.** Schematic illustration of the testing system. Subtle pressure stimuli were applied by (a) a mechanical testing stage equipped with a force sensor with an ultrasensitive force gauge and (b) a sound source.


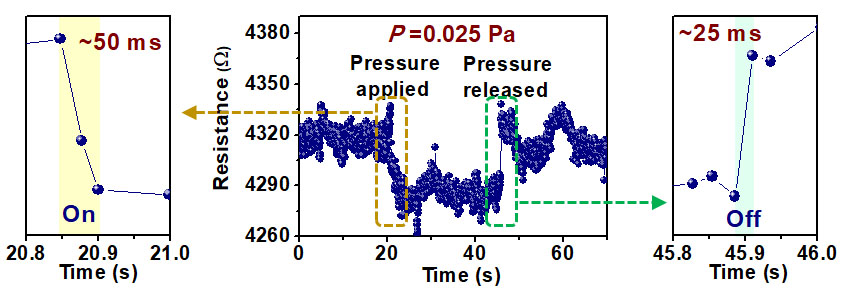


**Supplementary Figure 22.** Transient response to the application and removal of a pressure of 0.025 Pa on the BBP-MX-AG sensor.


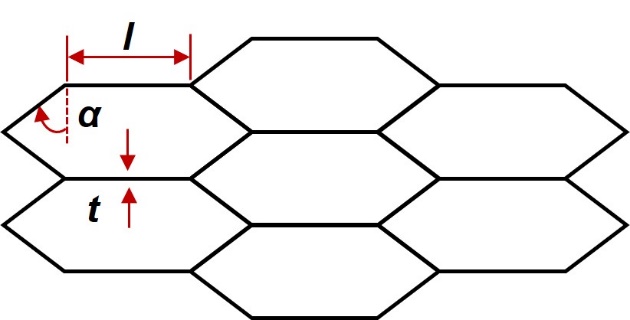


**Supplementary Figure 23.** The simplified microcell model for the MXene-based aerogel with a honeycomb-like porous structure for theoretical estimation.


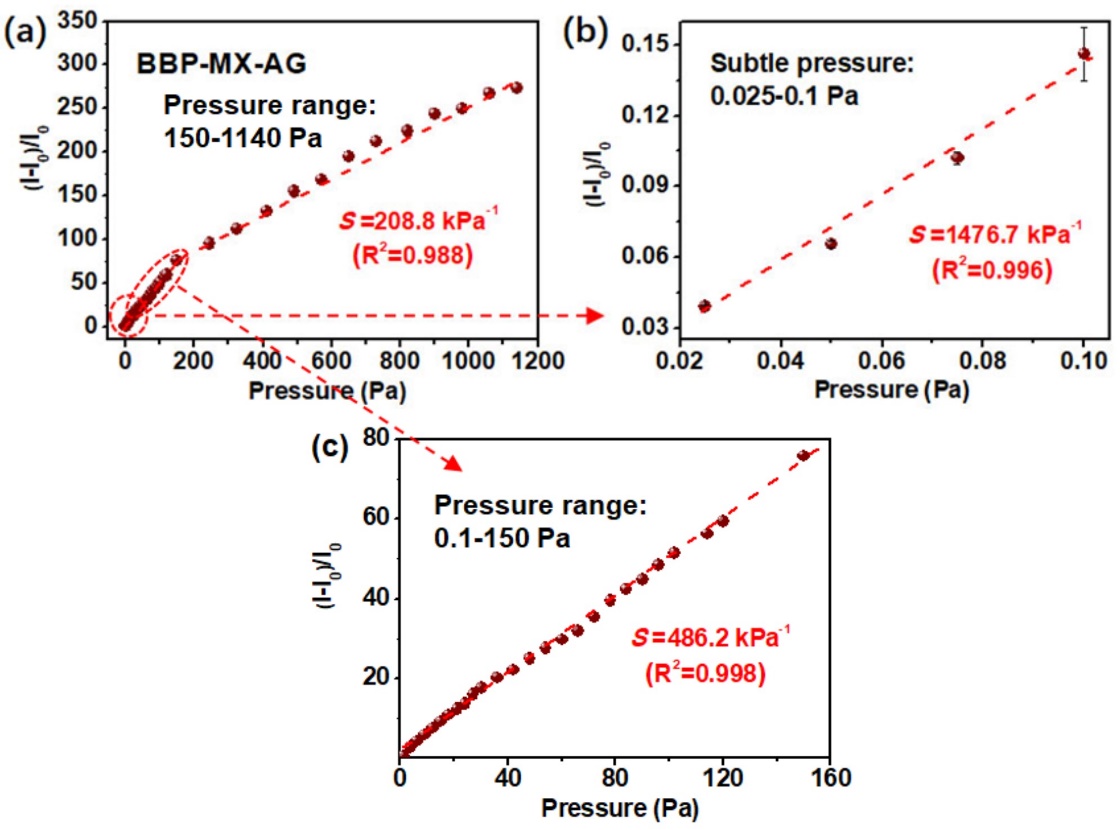


**Supplementary Figure 24.** Relative current changes of the BBP-MX-AG sensor under (a) full pressure range of 0–1150 Pa, (b) a subtle pressure range of 0.025–0.1 Pa and (c) a medium pressure range of 0.1-150 Pa. The pressure stimuli were supplied by a mechanical testing stage. The sensing curve can be divided into three pressure ranges of 0.025-0.1 Pa, 0.1-150 Pa, and 150-1140 Pa. The corresponding sensitivity under 0.025-0.1 Pa, 0.1-150 Pa, and 150-1140 Pa was 1476.7 kPa^-1^ with a linearity (*R*^2^) of 0.996, 486.2 kPa^-1^ with a linearity of 0.998, and 208.8 kPa^-1^ with a linearity of 0.988, respectively.


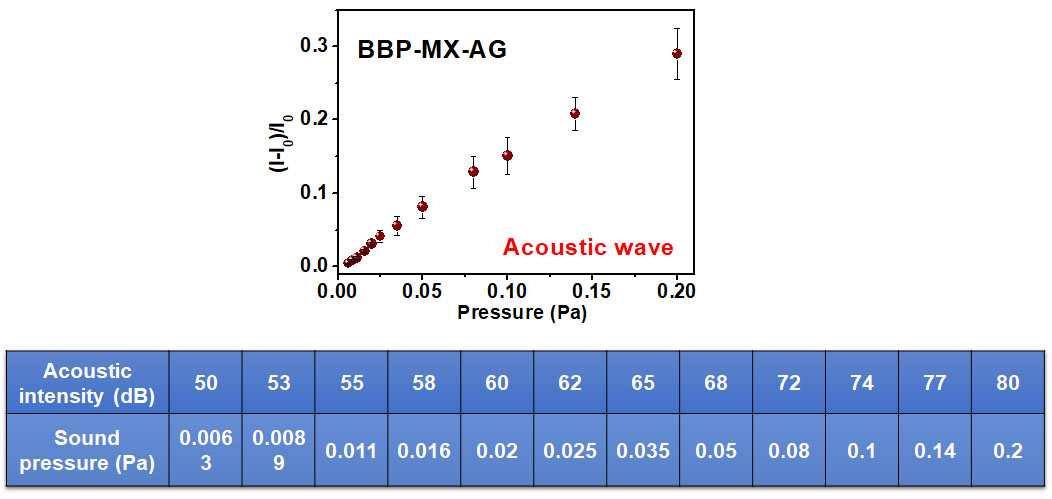


**Supplementary Figure 25.** Relative current changes of the BBP-MX-AG sensor towards a subtle pressure in the range of 0.0063–0.2 Pa. The pressure stimuli were supplied by a sound source with increasing acoustic intensities from 50 to 80 dB.


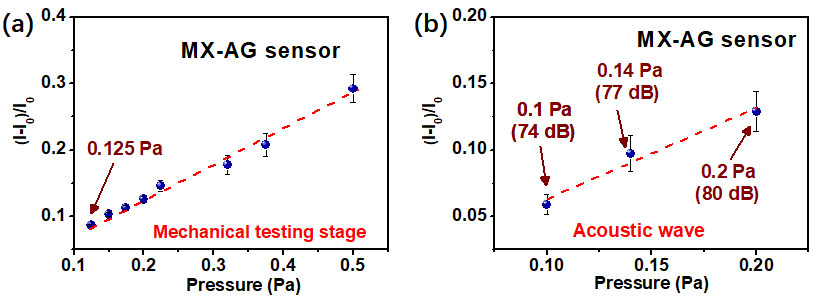


**Supplementary Figure 26.** Relative current changes of the MX-AG sensor under subtle pressures supplied by (a) a mechanical testing stage and (b) sound source. The minimum detectable pressure limit for the MX-AG sensor was ~0.1 Pa.


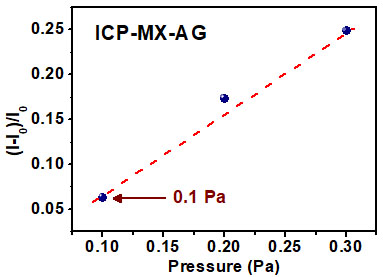


**Supplementary Figure 27.** Relative current changes of the ICP-MX-AG sensor under subtle pressure stimuli. The minimum detectable pressure limit for the ICP-MX-AG sensor was ~0.1 Pa.


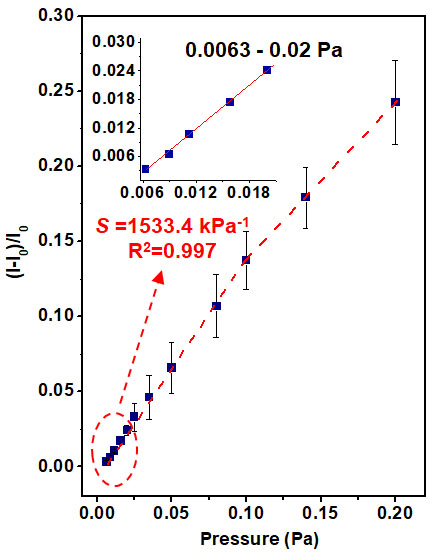


**Supplementary Figure 28.** Subtle pressure sensitivity of the BBP-MX-AG (with a mass density of ~7 mg/cm^3^) sensor stimulated by acoustic waves. The pressure detection limit was 0.0063 Pa (50 dB), and the pressure sensitivity was 1533.4 kPa^-1^ under a pressure range of 0.0063–0.02 Pa.


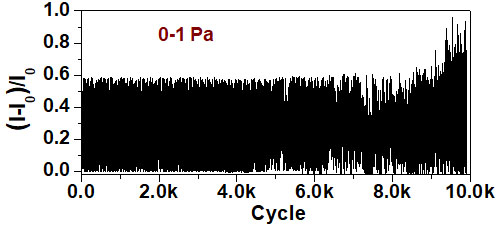


**Supplementary Figure 29.** Relative current changes of the MX-AG sensor over 10,000 compression-release cycles between 0–1 Pa.


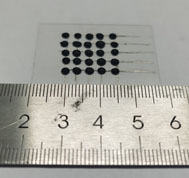


**Supplementary Figure 30.** Photograph of a 5 × 5 pixel sensing array with an active pixel area of 2 mm (diameter) and a total device area of 1.5 × 1.5 cm^2^.

**Supplementary Table 1.** Comparison of the minimum detectable pressure limit and corresponding sensitivity at a minimum pressure of the BBP-MX-AG piezoresistive sensor and previously reported state-of-the-art pressure sensors.

| Mechanisms | Materials | Structure | Minimum detectable limit (Pa) | Sensitivity^a^ (kPa^-1^) | Reference |
| --- | --- | --- | --- | --- | --- |
| **Piezoresistive** | **BBP-MXene** | **Aerogel with nanochannels** | **0.0063** | **1929.8** | **This work** |
| Piezoresistive | Zinc oxide microparticles | Sea urchin-shaped microparticles | 0.1 | 94 | 12 |
| Piezoresistive | Gold nanowires | Microstructured | 13 | 1.14 | 13 |
| Piezoresistive | Aligned carbon nanotubes/graphene | Microstructured | 0.6 | 19.8 | 14 |
| Piezoresistive | Ultrathin film of SWCNTs | Microstructured | 0.6 | 1.8 | 15 |
| Piezoresistive | Elastic graphene-based cellular monoliths | Aerogel | 0.082 | 10 | 16 |
| Piezoresistive | Gold film | Microstructured | 10.4 | 50.17 | 17 |
| Piezoresistive | Monolayer graphene | Hierarchically structured | 1 | 8.5 | 18 |
| Piezoresistive | Conducting MOFs nanocrystal arrays | Nanostructured | 0.73 | 6.25 | 19 |
| Piezoresistive | MXene-PVA | Sponge | 9 | 147 | 20 |
| Piezoresistive | MXene/tissue paper | Porous-structured | 10.2 | 0.55 | 21 |
| Piezoresistive | MXene/rGO | Aerogel | 10 | 4.05 | 22 |
| Piezoresistive | MXene/natural microcapsule | Microstructured | 8 | 24.63 | 23 |
| Piezoresistive | MXene/cellulose nanocrystal | Aerogel | 1.0 | 114.6 | 24 |
| Piezoresistive | MXene/chitosan | Aerogel | 1.0 | 21.5 | 25 |
| Piezoresistive | MXene/bacterial cellulose fiber | Aerogel | 1.0 | 81.7 | 26 |
| Piezoresistive | Polypyrrole | Porous-structured | 0.8 | 56 | 27 |
| Piezoresistive | Carbon nanotubes/PDMS | Microstructured | 0.2 | 15.1 | 28 |
| Piezoresistive | rGO | Microstructured | 1.5 | 5.5 | 29 |
| Piezoresistive | Graphene | Porous-structured | 9 | 161.6 | 30 |
| Piezoresistive | Zinc oxide | Micro- and nanostructured | 0.6 | 6.8 | 31 |
| Piezoresistive | Carbon black/TPU | Porous-structured | 10 | 5.54 | 32 |
| Piezoresistive | Metal nanoparticles/PU | Microstructured | 4 | 71.37 | 33 |
| Piezoresistive | rGO-molecular pillars | Microstructured | 7 | 0.82 | 34 |
| Piezoresistive | MXene interlayer | Micro- and nanostructured | 351 |  | 35 |
| Piezoresistive | MXene | Micro- and nanostructured | 9 | 99.5 | 36 |
| Piezoresistive | Carbon nanotubes/biodegradable organic materials | Microstructured | 12 | 0.7 | 37 |
| Piezoresistive | Urchin-like hollow carbon spheres | Micro- and nanostructured | 1 | 260.3 | 38 |
| Piezoresistive | Silver nanowire | Microstructured | 5 | 98000 | 39 |
| Piezoresistive | PEDOT: PSS | Microstructured | 0.025 | 380000 | 40 |
| Transistor-based piezoresistive | Organic thin-film transistors | Microstructured drain/source electrode | 10 | 113 | 41 |
| Transistor-based | Organic thin-film transistors | Suspended gate | 0.05 | 162.8 | 42 |
| Transistor-based capacitive | 2D semiconductor transistors | Microstructured air-gap gate | 0.08 | 100 | 43 |
| Piezo capacitive | PDMS | Microstructured | 6 | 0.62 | 44 |
| Iontronic | PVA/H_3_PO_4_ | Microstructured | 0.08 |  | 45 |

^a^ Sensitivity at the minimum detectable pressure limit.

**Supplementary Movie 1**

Dynamic in-situ HRTEM showing the reversible shrinking and expansion of nanochannels in the cellular walls of BBP-MX-AG.

**References.**

1. Zhang Q*, et al.* Hyperbolically Patterned 3D Graphene Metamaterial with Negative Poisson's Ratio and Superelasticity. *Adv. Mater.* **28**, 2229-2237 (2016).

2. Kilikevičius S, Kvietkaitė S, Žukienė K, Omastová M, Aniskevich A, Zeleniakienė D. Numerical investigation of the mechanical properties of a novel hybrid polymer composite reinforced with graphene and MXene nanosheets. *Comp. Mater. Sci.* **174**, 109497 (2020).

3. Lipatov A*, et al.* Elastic properties of 2D Ti_3_C_2_T*_x_* MXene monolayers and bilayers. *Sci. Adv.* **4**, eaat0491 (2018).

4. Plimpton S. Fast Parallel Algorithms for Short-Range Molecular Dynamics. *J. of Comput. Phys.* **117**, 1-19 (1995).

5. Senftle TP*, et al.* The ReaxFF reactive force-field: development, applications and future directions. *Npj Comput. Mater.***2**, 1-14 (2016).

6. Hong S, Van Duin ACT. Molecular Dynamics Simulations of the Oxidation of Aluminum Nanoparticles using the ReaxFF Reactive Force Field. *J. of Phys. Chem. C* **119**, 17876–17886 (2015).

7. Fang Q*, et al.* FTIR and XPS investigation of Er-doped SiO_2_–TiO_2_ films. *Mater. Sci. Eng.* **105**, 209-213 (2003).

8. Kumar S, Lei Y, Alshareef NH, Quevedo-Lopez M, Salama KN. Biofunctionalized two-dimensional Ti_3_C_2_ MXenes for ultrasensitive detection of cancer biomarker. *Biosens. Bioelectron.* **121**, 243-249 (2018).

9. Zhao J*, et al.* A hydrophobic surface enabled salt-blocking 2D Ti_3_C_2_ MXene membrane for efficient and stable solar desalination. *J. Mater Chem. A,* **6**, 16196-16204 (2018).

10. Cao W-T*, et al.* Two-dimensional MXene-reinforced robust surface superhydrophobicity with self-cleaning and photothermal-actuating binary effects. *Mater. Horiz.* **6**, 1057-1065 (2019).

11. Rasalingam S*, et al.* Influence of Ti–O–Si hetero-linkages in the photocatalytic degradation of Rhodamine B. *Catal. Commun.* **31**, 66-70 (2013).

12. Yin B, Liu X, Gao H, Fu T, Yao J. Bioinspired and bristled microparticles for ultrasensitive pressure and strain sensors. *Nat. Commun.* **9**, 1-8 (2018).

13. Gong S*, et al.* A wearable and highly sensitive pressure sensor with ultrathin gold nanowires. *Nat. Commun.* **5**, 1-8 (2014).

14. Jian M*, et al.* Flexible and highly sensitive pressure sensors based on bionic hierarchical structures. *Adv. Funct. Mater.* **27**, 1606066 (2017).

15. Wang X, Gu Y, Xiong Z, Cui Z, Zhang T. Silk‐molded flexible, ultrasensitive, and highly stable electronic skin for monitoring human physiological signals. *Adv. Mater.***26**, 1336-1342 (2014).

16. Qiu L*, et al.* Ultrafast Dynamic Piezoresistive Response of Graphene‐Based Cellular Elastomers. *Adv. Mater.* **28**, 194-200 (2016).

17. Su B, Gong S, Ma Z, Yap LW, Cheng W. Mimosa‐Inspired Design of a Flexible Pressure Sensor with Touch Sensitivity. *Small* **11**, 1886–1891 (2015).

18. Bae GY*, et al.* Linearly and highly pressure‐sensitive electronic skin based on a bioinspired hierarchical structural array. *Adv. Mater.* **28**, 5300-5306 (2016).

19. Fu X, Dong H, Zhen Y, Hu W. Solution‐Processed Large‐Area Nanocrystal Arrays of Metal–Organic Frameworks as Wearable, Ultrasensitive, Electronic Skin for Health Monitoring. *Small* **11**, 3351 (2015).

20. Yue Y*, et al.* 3D hybrid porous Mxene-sponge network and its application in piezoresistive sensor. *Nano Energy* **50**, 79-87 (2018).

21. Guo Y, Zhong M, Fang Z, Wan P, Yu G. A wearable transient pressure sensor made with MXene nanosheets for sensitive broad-range human–machine interfacing. *Nano Lett.* **19**, 1143-1150 (2019).

22. Ma Y*, et al.* 3D synergistical MXene/reduced graphene oxide aerogel for a piezoresistive sensor. *ACS Nano* **12**, 3209-3216 (2018).

23. Wang K*, et al.* Bioinspired interlocked structure-induced high deformability for two-dimensional titanium carbide (MXene)/natural microcapsule-based flexible pressure sensors. *ACS Nano* **13**, 9139-9147 (2019).

24. Zhuo H*, et al.* A carbon aerogel with super mechanical and sensing performances for wearable piezoresistive sensors. *J. Mater. Chem. A,* **7**, 8092-8100 (2019).

25. Hu Y*, et al.* Biomass polymer-assisted fabrication of aerogels from MXenes with ultrahigh compression elasticity and pressure sensitivity. *J. Mater. Chem. A,* **7**, 10273-10281 (2019).

26. Chen Z*, et al.* Compressible, elastic, and pressure-sensitive carbon aerogels derived from 2D titanium carbide nanosheets and bacterial cellulose for wearable sensors. *Chem. Mater.* **31**, 3301-3312 (2019).

27. Pan L*, et al.* An ultra-sensitive resistive pressure sensor based on hollow-sphere microstructure induced elasticity in conducting polymer film. *Nat. Commun.* **5**, 1-8 (2014).

28. Park J*, et al.* Giant Tunneling Piezoresistance of Composite Elastomers with Interlocked Microdome Arrays for Ultrasensitive and Multimodal Electronic Skins. *ACS Nano* **8**, 4689-4697 (2014).

29. Zhu B, Niu Z, Wang H, Wan RL, Chen X. Microstructured Graphene Arrays for Highly Sensitive Flexible Tactile Sensors. *Small* **10**, 3625-3631 (2014).

30. Sheng L*, et al.* Bubble‐decorated honeycomb‐like graphene film as ultrahigh sensitivity pressure sensors. *Adv. Funct. Mater.* **25**, 6545-6551 (2015).

31. Ha M, Lim S, Park J, Um DS, Lee Y, Ko H. Bioinspired interlocked and hierarchical design of ZnO nanowire arrays for static and dynamic pressure‐sensitive electronic skins. *Adv. Funct. Mater.* **25**, 2841-2849 (2015).

32. Wang Z, Guan X, Huang H, Wang H, Lin W, Peng Z. Full 3D printing of stretchable piezoresistive sensor with hierarchical porosity and multimodulus architecture. *Adv. Funct. Mater.* **29**, 1807569 (2019).

33. Lee D, Kim J, Kim H, Heo H, Park K, Lee Y. High-performance transparent pressure sensors based on sea-urchin shaped metal nanoparticles and polyurethane microdome arrays for real-time monitoring. *Nanoscale* **10**, 18812-18820 (2018).

34. Huang CB*, et al.* Molecule–graphene hybrid materials with tunable mechanoresponse: highly sensitive pressure sensors for health monitoring. *Adv. Mater.* **31**, 1804600 (2019).

35. Ma Y*, et al.* A highly flexible and sensitive piezoresistive sensor based on MXene with greatly changed interlayer distances. *Nat. Commun.* **8**, 1207 (2017).

36. Gao Y*, et al.* Microchannel‐Confined MXene Based Flexible Piezoresistive Multifunctional Micro‐Force Sensor. *Adv. Funct. Mater.* **30**, 1909603 (2020).

37. Boutry CM*, et al.* A stretchable and biodegradable strain and pressure sensor for orthopaedic application. *Nat. Electron.* **1**, 314-321 (2018).

38. Shi L, Li Z, Chen M, Qin Y, Wu L. Quantum effect-based flexible and transparent pressure sensors with ultrahigh sensitivity and sensing density. *Nat. Commun.* **11**, (2020).

39. Pu J-H*, et al.* Human skin-inspired electronic sensor skin with electromagnetic interference shielding for the sensation and protection of wearable electronics. *ACS Appl. Mater. Inter.* **10**, 40880-40889 (2018).

40. Lee Y*, et al.* Bioinspired Gradient Conductivity and Stiffness for Ultrasensitive Electronic Skins. *ACS Nano* **15**, 1795-1804 (2020).

41. Wang Z*, et al.* The semiconductor/conductor interface piezoresistive effect in an organic transistor for highly sensitive pressure sensors. *Adv. Mater.* **31**, 1805630 (2019).

42. Zang Y, Zhang F, Huang D, Gao X, Di CA, Zhu D. Flexible suspended gate organic thin-film transistors for ultra-sensitive pressure detection. *Nat. Commun.* **6**, 6269 (2015).

43. Huang YC, Liu Y, Ma C, Cheng HC, Duan X. Sensitive pressure sensors based on conductive microstructured air-gap gates and two-dimensional semiconductor transistors. *Nat. Electron.* **3**, 59-69 (2020).

44. Lee K*, et al.* Rough‐Surface‐Enabled Capacitive Pressure Sensors with 3D Touch Capability. *Small* **13**, 1700368 (2017).

45. Bai N*, et al.* Graded intrafillable architecture-based iontronic pressure sensor with ultra-broad-range high sensitivity. *Nat. Commun.* **11**, 1-9 (2020).
