# Supplementary material for: Pushing detectability and sensitivity for subtle force to new limits with shrinkable nanochannel structured aerogel
Source: Nat Commun. 2022 Mar 2;13:1119. doi: 10.1038/s41467-022-28760-4 (PMC8891261; doi:10.1038/s41467-022-28760-4)
Supplement: Supplementary file 3 — Description of Additional Supplementary Files [file 41467_2022_28760_MOESM3_ESM.pdf]

Description of Additional Supplementary Files

File name: Supplementary Movie 1

Description: Dynamic in-situ HRTEM showing the reversible shrinking and expansion of nanochannels in the cellular walls of BBP-MX-AG.
